# A Comprehensive Multi-Omics Study of Serum Alterations in Red Deer Infected by the Liver Fluke *Fascioloides magna*

**DOI:** 10.3390/pathogens13110922

**Published:** 2024-10-22

**Authors:** Josipa Kuleš, Miljenko Bujanić, Ivana Rubić, Karol Šimonji, Dean Konjević

**Affiliations:** 1 Department of Chemistry and Biochemistry, Faculty of Veterinary Medicine, University of Zagreb, 10000 Zagreb, Croatia; 2 Educational Center for Game Management I/3 “Črnovšćak”, Faculty of Veterinary Medicine, University of Zagreb, 10000 Zagreb, Croatia; mbujanic@vef.unizg.hr; 3 Internal Diseases Clinic, Faculty of Veterinary Medicine, University of Zagreb, 10000 Zagreb, Croatia; irubic@vef.unizg.hr (I.R.); ksimonji@vef.unizg.hr (K.Š.); 4 Department of Veterinary Economics and Epidemiology, Faculty of Veterinary Medicine, University of Zagreb, 10000 Zagreb, Croatia; konjevic@vef.unizg.hr

**Keywords:** proteomics, metabolomics, wildlife, host–pathogen interaction, liver fluke

## Abstract

Liver fluke infections are acknowledged as diseases with global prevalence and significant implications for both veterinary and public health. The large American liver fluke, *Fascioloides magna*, is a significant non-native parasite introduced to Europe, threatening the survival of local wildlife populations. The aim of this study was to analyze differences in the serum proteome and metabolome between *F. magna*-infected and control red deer. Serum samples from red deer were collected immediately following regular hunting operations, including 10 samples with confirmed *F. magna* infection and 10 samples from healthy red deer. A proteomics analysis of the serum samples was performed using a tandem mass tag (TMT)-based quantitative approach, and a metabolomics analysis of the serum was performed using an untargeted mass spectrometry-based metabolomics approach. A knowledge-driven approach was applied to integrate omics data. Our findings demonstrated that infection with liver fluke was associated with changes in amino acid metabolism, energy metabolism, lipid metabolism, inflammatory host response, and related biochemical pathways. This study offers a comprehensive overview of the serum proteome and metabolome in response to *F. magna* infection in red deer, unveiling new potential targets for future research. The identification of proteins, metabolites, and related biological pathways enhances our understanding of host–parasite interactions and may improve current tools for more effective liver fluke control.

## 1. Introduction

For many years, wild animals have been reported as a potential source of infectious and parasitic diseases for domestic animals and humans [1,2]. It is evident that the emphasis has been on preserving the health of humans and domestic animals, while the impact of pathogens on wildlife has generally been a secondary concern, likely due to past perspectives shaped by economic considerations and public health policies. The shift to the integrated One Health approach recognizes that the health of humans, animals (including wildlife), and ecosystems is interconnected and interdependent. Additionally, the increasing role of emerging diseases in the wildlife population is well recognized [3]. Numerous factors, including invasive species, climate change, pollution, and resource overexploitation, negatively affect wildlife population health [4].

Parasites can affect the behavior, health, and population dynamics of wildlife, and their presence in wild populations is often used as an indicator of ecosystem health. Introducing non-native parasites to naïve hosts can result in losses in livestock production and negatively impact the survival of local wildlife populations [1]. The recent rising prevalence of fascioloidosis in Central Europe is an example of a non-native parasite being introduced to naïve hosts. The giant liver fluke, *Fascioloides magna* (Digenea: *Fasciolidae*), is originally a parasite of North American deer species, introduced to Europe through infected white-tailed (*Odocoileus virginianus*) and wapiti deer (*Cervus elaphus canadensis*) [5,6]. Fascioloidosis, a parasitic disease caused by the non-native parasite *F. magna*, has significant implications for wild and domestic animals, namely the management and conservation of game and domestic animal husbandry.

The life cycle of *F. magna* begins with mature flukes releasing eggs, which are then excreted in the feces of the mammalian host [6]. In water, miracidia hatch from the eggs and actively seek out an aquatic snail, the intermediate host. Within the snail, the parasite undergoes several developmental stages: sporocysts, rediae, and cercariae. The cercariae then leave the snail and encyst as metacercariae on vegetation becoming an infective stage. Once ingested by a mammalian host, the metacercariae excyst, and the juvenile flukes penetrate the intestinal walls, migrate through the peritoneal cavity to reach the liver, penetrate the Glisson’s capsule, and migrate through the liver parenchyma. Finally, juvenile flukes encapsulate within pseudocysts and develop into mature flukes.

The pathological changes, clinical signs, and outcomes of *F. magna* infection are strongly related to the three types of final hosts—definitive, aberrant and dead-end—and their different tolerance to infection [5,6]. In definitive hosts like red deer, the parasite reaches its sexual maturity placed within a pseudocyst in the liver parenchyma, and animals rarely show any clinical signs [7,8]. However, due to egg shedding through feces and their large migration potential, red deer as definitive hosts are extremely important for the maintenance and spread of *F. magna* in European nature.

The latest advances in omics approaches, particularly in relation to genomic and transcriptomic sequencing, have provided important tools for the characterization of pathogen proteomes. Proteomics, as the study of the proteome, involves connecting genes with their functionally diverse protein products. Recently, there have been significant efforts to identify and characterize the host–pathogen molecular interface, with particular emphasis on secretomes and surface proteomes from blood and liver flukes, as well as nematodes [9,10,11,12,13,14,15].

For the fluke *F. magna*, the transcriptome and the excretory and secretory proteome of sexually mature flukes, obtained from naturally infected red deer livers, were characterized, setting up a foundation for future research [16]. A comparative proteomics analysis of the liver and serum of wild boar, a dead-end host for *F. magna* infection, provided insights into proteome profile changes at both local and systemic levels [17]. This study revealed associations with host immune response, oxidative stress, and metabolism changes. A recent proteomics study also revealed alterations in the metabolism of proteins and fatty acids, as well as modifications related to oxidative stress, fibrosis, and metabolism signaling pathways in the liver of red deer infected by *F. magna* [18]. More studies can be found related to infections with liver fluke *Fasciola hepatica* and *Fasciola gigantica* [19,20].

The metabolic effects of parasites on the host were studied before the ‘omics’ era. Parasites and hosts compete for energy resources and, therefore, this profoundly affects metabolic homeostasis. With the emergence of the new omics discipline of metabolomics, parasitic helminth infections were among the first experimental models to which it was applied [21,22]. Metabolomics involves the high-throughput characterization of metabolites, small molecular compounds (<1.5 kDa) that are the end products of the cellular metabolism and serve as direct indicators of biochemical activity [23].

Integrated omics approaches are required to obtain much deeper insights than any of these techniques alone, providing a more comprehensive understanding of the molecular mechanisms involved in disease development [24]. Combining individual omics data, such as proteomics and metabolomics, emphasizes the relationships between the involved biomolecules and their functions, and furthermore helps bridge the gap from genotype to phenotype [25].

The aim of this study was to analyze differences in serum proteome between *F. magna*-infected and apparently healthy red deer (*Cervus elaphus*) that served as the control group, using a label-based high-resolution liquid chromatography–tandem mass spectrometry (LC-MS/MS) quantitative proteomics approach. Furthermore, an untargeted MS-based metabolomics approach was employed to assess differences in the serum metabolome. The bioinformatic functional analysis of proteins and metabolites with varying abundances and intensities facilitated the understanding of the mechanisms underlying host–parasite interactions. A knowledge-driven approach was applied to map data from each omics layer to curated knowledge bases containing annotations in terms of pathways and interactions for different types of biomolecules.

## 2. Materials and Methods

### 2.1. Animals

Serum samples were collected immediately following regular hunting operations. Blood samples were obtained from large veins or the heart using a syringe. Samples were centrifuged using a portable centrifuge (Hettich, EBA 20, Merck KGaA, Darmstadt, Germany), the serum was transferred into tubes, properly labeled and frozen at −80 °C until further analysis. The health status of the red deer was assessed based on external appearance, body condition, fur appearance, and behavior before shooting, and afterward, based on the presence of any visible wounds, discharge from natural openings, and the macroscopic appearance of the organs during evisceration. Age was determined according to body development and tooth characteristics. All animals were females and young adults older than 2 years.

A parasitological examination of the liver was carried out for fascioloidosis diagnosis [26]. Liver slices (approx. 2 cm thick) were thoroughly examined for traces of iron-porphyrin, fluke migratory paths, pseudocysts, and juvenile or adult flukes. In positive animals, numerous infection-specific features were present, while the livers of uninfected control animals appeared normal, free of *F. magna* flukes, and without any related gross lesions.

All animals had lungworms and strongyles. Considering that this is a common finding in red deer and does not cause any symptoms, the only difference between the control and case groups was the presence of the liver fluke *F. magna*. Thus, we hypothesize that the observed alterations in the serum proteome and metabolome are attributable to the *F. magna* infection.

In total, the study involved 20 animals, including 10 animals that served as the control group and 10 animals with confirmed *F. magna* infection. The study was approved by the Committee on the Ethics of the University of Zagreb, Faculty of Veterinary Medicine (Class: 640-01/18-17/60, Reg. number: 251-61-44-18-02).

### 2.2. Proteomics Analysis

#### 2.2.1. Sample Preparation and LC-MS/MS Analysis

A proteomics analysis of the serum samples was performed using a tandem mass tag (TMT)-based quantitative approach, as described previously [27]. Total protein concentration was measured with a BCA assay (Thermo Scientific, Rockford, IL, USA). Thirty-five μg of total proteins were diluted to a volume of 50 μL using 0.1 M triethyl ammonium bicarbonate (TEAB, Thermo Scientific, Rockford, IL, USA), reduced (200 mM DTT (Sigma Aldrich, St. Louis, MO, USA), 60 min, 55 °C), alkylated (375 mM IAA (Sigma Aldrich, St. Lois, MO, USA), 30 min, room temperature in the dark) and acetone-precipitated (overnight, −20 °C). After centrifugation (9000× *g*, 4 °C), the protein pellet was dissolved in 0.1 M TEAB and digested using 1 μL of trypsin (1 mg/mL, (Promega, Madison, WI, USA); trypsin-to-protein ratio 1:35, at 37 °C overnight). Samples and internal standard were labeled with TMT 6plex reagents (Thermo Scientific, Rockford, IL, USA) according to the manufacturer’s procedure.

LC-MS/MS analysis of TMT-labeled peptides was carried out using an Ultimate 3000 RSLCnano system (Dionex, Germering, Germany) coupled to a Q Exactive Plus mass spectrometer (Thermo Fisher Scientific, Bremen, Germany). After dissolving in the loading solvent (2% acetonitrile (ACN), 0.1% formic acid), TMT-labeled peptides were desalted on the trap column (C18 PepMap100, 5 µm, 100 A, 300 µm × 5 mm; Thermo Fisher Scientific, Waltham, MA, USA) for 12 min at a flow rate of 15 µL/min. Mobile phase A consisted of 0.1% formic acid in water, and mobile phase B was 0.1% formic acid in 80% ACN. Separation was performed on the analytical column (PepMap™ RSLC C18, 50 cm × 75 μm; Thermo Fisher Scientific, Waltham, MA, USA) using a linear gradient of 5–55% mobile phase B over 120 min, followed by an increase to 95% for 1 min, held at 95% for 2 min, and re-equilibrated at 5% B for 20 min at a flow rate of 300 nL/min. Ionization was achieved using a nanospray Flex ion source (Thermo Fisher Scientific, Bremen, Germany) with a SilicaTip emitter with an inner diameter of 10 μm (New Objective, Woburn, MA, USA). The MS operated in positive ion mode using the DDA Top8 method. Full-scan MS spectra were acquired in the range from *m*/*z* 350.0 to *m*/*z* 1800.0 with a resolution of 70,000, 120 ms injection time, AGC target 1 × 10^6^, ± 2.0 Da isolation window, and a dynamic exclusion of 30 s. High-energy collisional dissociation (HCD) fragmentation was performed at step collision energy (29% and 35% NCE) at a resolution of 17,500 and an AGC target of 2 × 10^5^. Precursor ions with an unassigned charge state, charge states of +1, or more than +7 were excluded from fragmentation.

#### 2.2.2. Data Processing

The acquired MS/MS spectra were analyzed for protein identification and quantification using the SEQUEST algorithm implemented in Proteome Discoverer (version 2.3., Thermo Fisher Scientific). A database search against *Cervus elaphus* FASTA files (downloaded from Uniprot database on 5 July 2022, containing 19,262 sequences) was performed using the following parameters: two trypsin missed cleavage sites, precursor mass tolerances of 10 ppm, a fragment mass tolerance of 0.02 Da, carbamidomethyl (C) as a fixed peptide modification, and oxidation (M) and TMT sixplex (K, peptide N-terminus) as dynamic modifications. The false discovery rate (FDR) for peptide identification was set at 1% and calculated using the Percolator algorithm in the Proteome Discoverer workflow. To report confidently identified proteins, at least two unique peptides and a 1% FDR were required. Protein abundances were normalized to the total protein amount and scaled to the control average to enable comparison within one sixplex and between different sixplexes, respectively.

#### 2.2.3. Statistical and Bioinformatic Analysis

Statistical analysis was performed using R software v.4.1.2. [28], following the previously published in-house protocol [17]. Differences between groups were tested using the non-parametric Mann–Whitney U test, with Benjamini–Hochberg false discovery rate (FDR) correction, after removing proteins with abundances missing in more than half of the samples. Principal component analysis (PCA) and volcano plots were generated using the R package ggplot2 v3.1.1 [29]. For bioinformatic analysis, protein accession numbers were converted to Gene IDs using the UniProt database conversion tool, and replaced with the *Bos taurus* orthologue if an identity higher than 70% was found using the UniProt BLAST tool [30]. Gene ontology (GO) classification was performed using the Protein Analysis Through Evolutionary Relationship (PANTHER) classification tool [31]. Pathway enrichment analysis was conducted with the Reactome tool, using the human genome as the background and an FDR-adjusted *p*-value < 0.05 to identify significantly enriched pathways [32].

#### 2.2.4. Validation of Proteomics Data

Frozen serum samples were thawed and analyzed using a sandwich enzyme-linked immunosorbent assay (ELISA) to determine alpha-1-acid glycoprotein levels. A cattle a1AGP (Alpha-1-Acid Glycoprotein) ELISA kit (ELK Biotechnology, Denver, CO, USA) was used according to the manufacturer’s instructions.

### 2.3. Untargeted Metabolomics Analysis

#### 2.3.1. Sample Preparation and LC-MS/MS Analysis

Serum samples for untargeted metabolomics analysis were prepared using protein precipitation, centrifugation, and supernatant filtration. For metabolite extraction, an extraction solvent consisting of a chloroform/methanol/water (1:3:1, *v*/*v*/*v*) mixture was used (chloroform, methanol (Honeywell, Charlotte, NC, USA), water (Merck, Darmstadt, Germany)). Briefly, 1000 µL of ice-cold extraction mixture was added to 25 µL of each serum sample and vortexed on a cooled mixer at 4 °C for 5 min. A pooled sample was prepared by mixing 10 µL of each individual sample, and 25 µL of the pooled sample was also subjected to extraction. All assayed samples (individual samples, pooled samples, matrix blank) were subsequently vortexed and centrifuged (13,000× *g* for 5 min at 4 °C).

The analysis of metabolite extracts was performed on a Dionex UltiMate 3000 UHPLC system (Thermo Fisher Scientific, Germering, Germany) coupled with a Thermo Orbitrap Q Exactive (Thermo Fisher Scientific, Bremen, Germany). The metabolites were separated using hydrophilic interaction liquid chromatography (HILIC) with a ZIC-pHILIC column (150 mm × 4.6 mm, 5 µm column, Merck Sequant, Darmstadt, Germany). Metabolites were eluted using a linear gradient from 80% of mobile phase B to 5% (mobile phase A: 20 mM ammonium carbonate in water; mobile phase B: 100% ACN), with a flow rate of 0.3 mL/min. The Orbitrap Q Exactive mass spectrometer was operated in both positive and negative modes at a mass resolution of 70,000, covering the full scan range of *m*/*z* 70-1050. The source voltage was set to 3.8 kV for positive mode and −3.8 kV for negative mode, with sheath gas at 40 (arbitrary units), auxiliary gas at 5 (arbitrary units), and a capillary temperature of 320 °C. A standard mix (kindly provided by Glasgow Polyomics, United Kingdom) consisted of 148 compounds (reference compounds for metabolite identification) and was analyzed alongside the samples.

#### 2.3.2. Data Processing

Metabolomics data pre-processing (e.g., alignment, batch correction, and identification) was performed using the Polyomics integrated Metabolomics Pipeline (PiMP) available at http://polyomics.mvls.gla.ac.uk (assessed on 7 February 2024), implemented as an R pipeline based around XCMS for the feature detection and mzMatch.R for common metabolomics data pre-processing tasks [33]. Metabolites were identified based on the retention times/masses or masses of detected peaks matched to the standards, while features were annotated using the metabolite databases HMDB (The Human Metabolome Database) and/or KEGG (Kyoto Encyclopedia of Genes and Genomes) integrated within the PiMP software.

#### 2.3.3. Statistical and Bioinformatic Analysis

All statistical and pathway enrichment analyses were performed using online available software MetaboAnalyst v.6.0 [34], on the combined positive and negative ion datasets exported as a peak intensity table from PiMP.

Missing values (1.3%) were replaced by 1/5 of the minimum positive value for each variable. The intensities of the extracted metabolites were normalized by median, log transformed and then mean centered and divided by the square root of the standard deviation of each variable (Pareto scaling). The differences in the metabolic profiles of two groups, control and *F. magna*-infected group, were assessed by *t*-test with FDR correction (*p* < 0.05). The RaMP-DB pathway-associated metabolite sets were selected as the pathway library for Metabolite Set Enrichment Analysis (MSEA).

### 2.4. Integration of Proteomics and Metabolomics Data

Significant proteins and metabolites identified between the control and *F. magna*-infected group were merged to form a network for visualization and functional analysis using OmicsNet [35]. A knowledge-driven multi-omics network was created and the InfoMap algorithm was selected to search for densely connected subnetworks (modules) by compressing the description of information flows based on random walks [36].

## 3. Results

### 3.1. Proteomics Analysis

**Figure 1:**
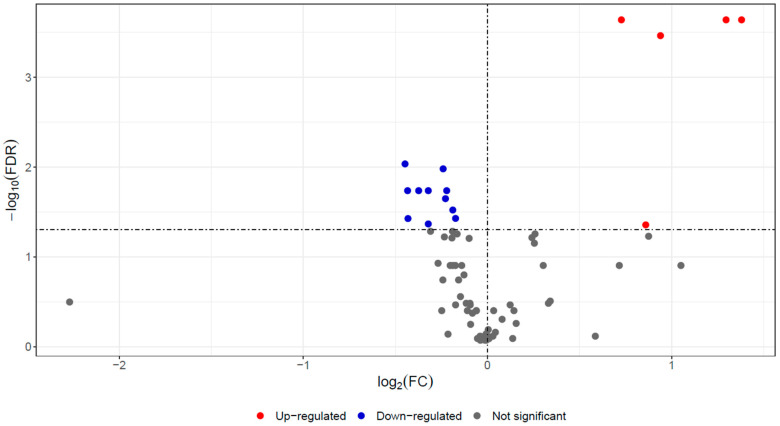
Volcano plot showing proteins with differential abundance in the *F. magna*-infected group (N = 10) compared to the control group (N = 10). Red points indicate significantly increased abundances in the infected group and blue points indicate significantly decreased protein abundances. Features that do not meet the significance threshold are shown in gray.

In serum samples of red deer, 76 proteins were identified and quantified using a label-based quantitative proteomic approach (Supplementary Table S1). Significant differences in abundances between the *F. magna*-infected and control groups were assessed by the Mann–Whitney test with FDR correction (FDR < 0.05), revealing differences for 18 proteins (Table 1). Of those, 5 exhibited higher abundance, and 13 showed lower abundance in the *F. magna*-infected group compared to the control group, as depicted in the volcano plot (Figure 1).

**Figure 2:**
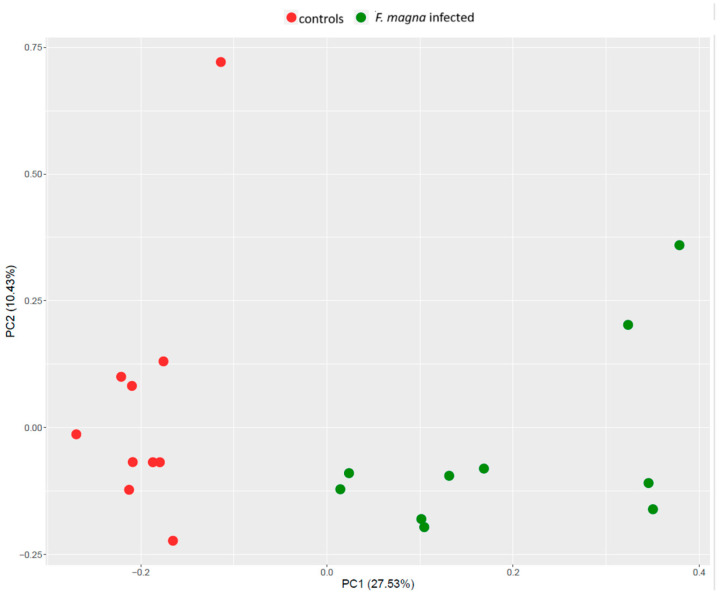
Principal component analysis (PCA) score plot showing the distribution of samples from the control (red dots) and *F. magna*-infected (green dots) group.

The PCA plot showed clear discrimination between the groups (Figure 2). The clusters were separated based on principal component 1 (PC1), which captured 27.5% of the variance in the dataset, while PC2 captured 10.4% of the variance.

**Figure 3:**
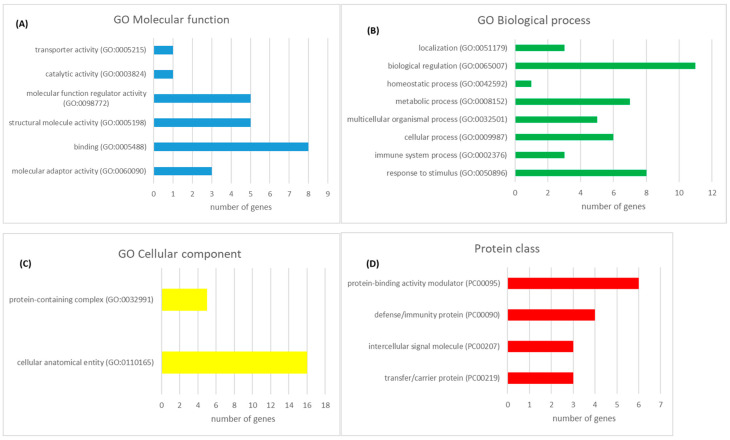
Gene ontology analysis for proteins with different abundance in serum between the control and *F. magna*-infected red deer using the PANTHER GO-Slim analysis: (**A**) molecular function; (**B**) biological process; (**C**) cellular component; (**D**) protein class.

According to PANTHER GO Slim analysis, serum proteins with significant differences in abundances between the control and *F. magna*-infected group were involved in biological regulation (N = 11), response to stimulus (N = 8), and metabolic processes (N = 7), among others (Figure 3). The molecular function of proteins with significantly differential abundances included binding (N = 8), molecular function regulator activity (N = 5), and structural molecule activity (N = 5).

**Figure 4:**
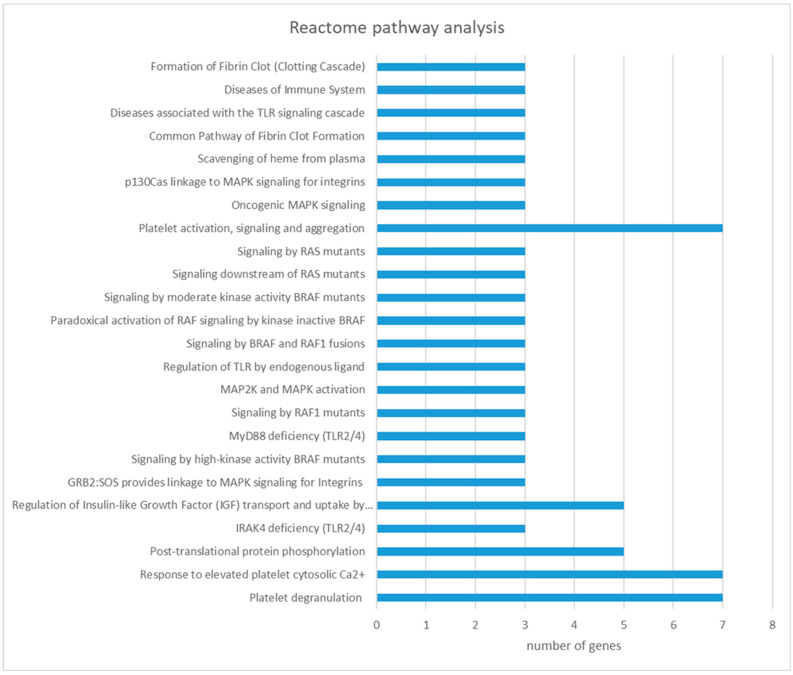
Reactome pathways enriched from serum proteins with differential abundance between the control and *F. magna*-infected red deer (FDR < 0.05).

Reactome pathway analysis revealed 24 significant pathways (Supplementary Table S2). Most of the significant serum proteins were involved in platelet degranulation (N = 7), response to elevated platelet cytosolic Ca^2+^ (N = 7), platelet activation, signaling and aggregation (N = 7), post-translational protein phosphorylation (N = 5), regulation of insulin-like growth factor (IGF) transport and uptake by insulin-like growth factor binding proteins (IGFBPs) (N = 5), and others (Figure 4).

Validating assays such as ELISA or Western blot for non-model species, like deer, poses significant challenges due to the specificity of antibodies. In the absence of cervid-specific kits, we employed a cattle-specific ELISA, given that cattle are the closest relatives to deer. Although we successfully generated a calibration curve using the kit’s standards, the deer serum samples did not produce a detectable signal. This highlights the limitations of using cross-species reagents and underscores the need for species-specific assay development in future studies.

### 3.2. Metabolomics Analysis

The metabolomics analysis resulted in the detection of 2744 features in serum samples of red deer (Supplementary Table S3). Among them, a total of 67 metabolites were identified based on mass/retention time matching with known standards (Supplementary Table S4).

**Figure 5:**
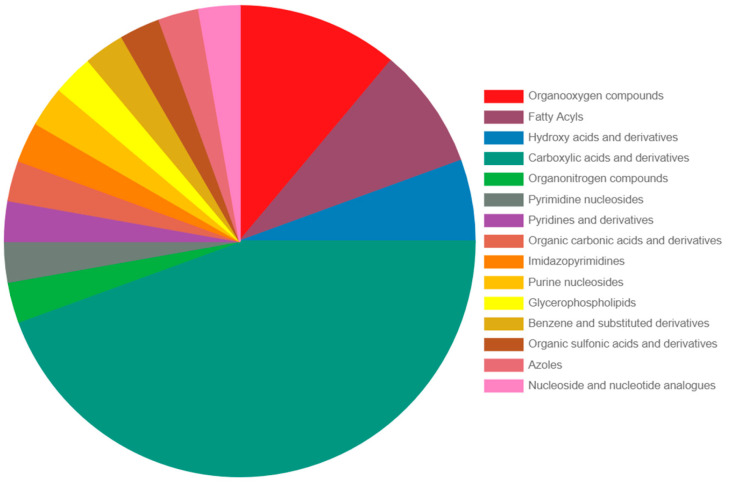
Distribution of main chemical classes of identified differential metabolites between the control and *F. magna*-infected red deer.

**Figure 6:**
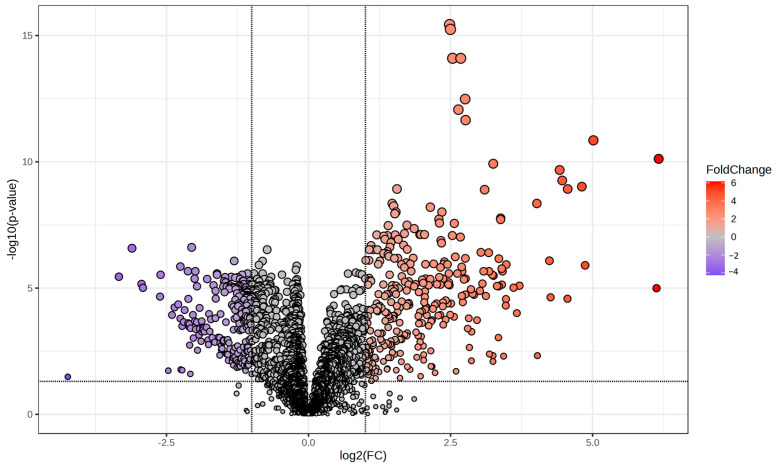
Volcano plot showing features with differential intensities between the control (N = 10) and *F. magna*-infected group (N = 10). Red points indicate significantly increased levels in the infected group and blue points indicate significantly decreased levels. Features that do not meet the significance threshold are shown in gray.

A *t*-test with FDR correction (*p* < 0.05) showed that 1789 features were significantly altered among the groups, while 44 metabolites were identified by reference to standards (Table 2). Most of these metabolites belonged to carboxylic acids, hydroxy acids and derivatives, organooxygen compounds, and fatty acyls (Figure 5). When applying a fold change threshold of 2.0, 329 features had higher intensity, and 224 had lower intensity in the *F. magna*-infected group compared to the control group, as depicted in the volcano plot (Figure 6).

**Figure 7:**
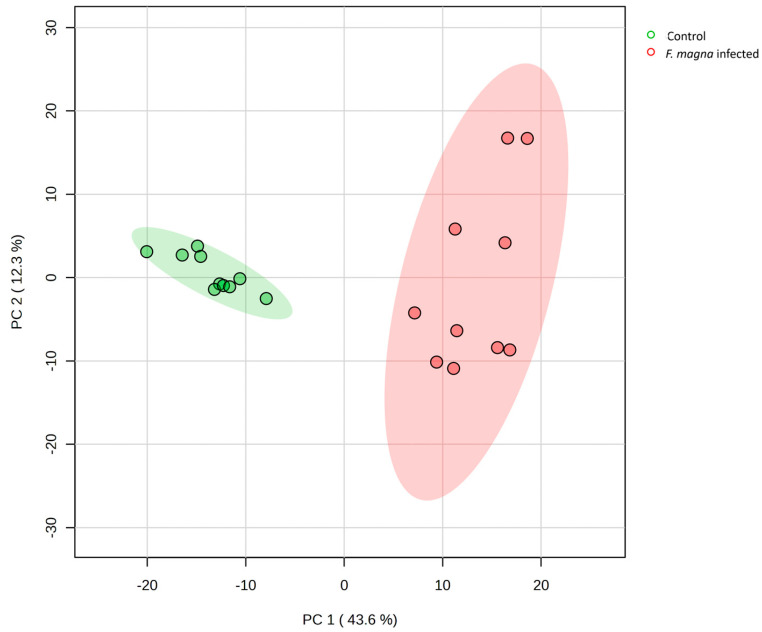
Principal component analysis (PCA) score plot showing the distribution of samples from the control group (green dots), and the *F. magna*-infected group (red dots), with the green and red backgrounds representing 95% confidence intervals.

Very clear discrimination of the groups is shown using PCA plot (Figure 7). The clusters were separated based on the principal component 1 (PC1), which captured 43.6% of the variance in the dataset, while PC2 captured 12.3% of the variance.

**Figure 8:**
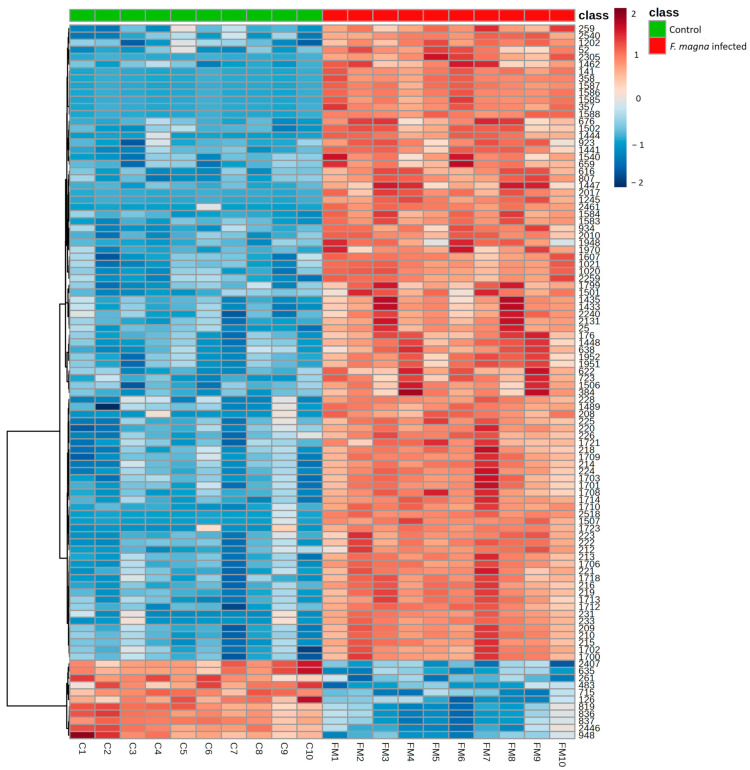
Hierarchical cluster analysis (HCA) based on the top 100 features with significantly differential intensities between the control (green panel) and *F. magna*-infected (red panel) group using Euclidean as a distance measure and Ward as a clustering algorithm. Each colored cell on the map corresponds to the intensity value, with the red color indicating increased, and blue a decreased level of a specific feature.

The distribution of the 100 most significant features separating the investigated groups was visualized by hierarchical cluster analysis using Euclidean distance as the measure, the Ward method as the clustering algorithm, and a *t*-test for feature ranking. The heat map showed a clear distinction between the control and infected group (Figure 8).

**Figure 9:**
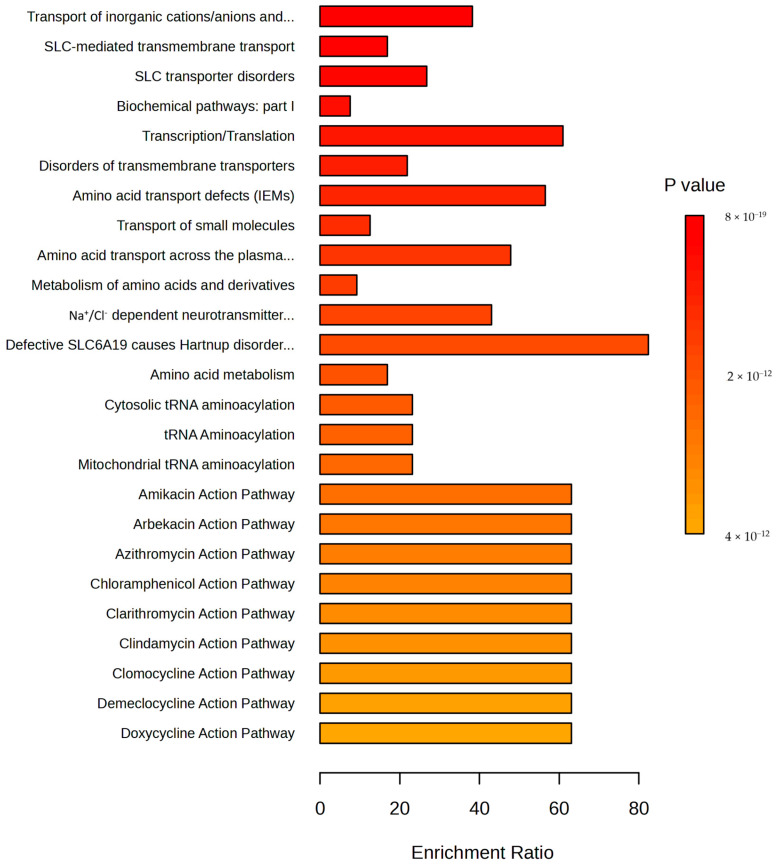
Pathway enrichment (top 25) of significant metabolites identified by the untargeted metabolomic approach between control and *F. magna*-infected group based on 3694 metabolites and lipid pathways from RaMP-DB.

To identify biological patterns associated with significant metabolites, pathway enrichment analysis was based on 3694 metabolites and lipid pathways from RaMP-DB (integrating KEGG via HMDB, Reactome and WikiPathways). The enrichment analysis revealed 184 significant pathways (FDR > 0.05) (Supplementary Table S5), while 25 of the most significant pathways are shown in Figure 9. Among these, pathways with the most identified metabolites included biochemical pathways—part I, the transport of small molecules, the metabolism of amino acids and derivatives, and SLC-mediated transmembrane transport. Of special interest were various antibiotic action pathways, which were also enriched.

### 3.3. Integration of Proteomics and Metabolomics Data

**Figure 10:**
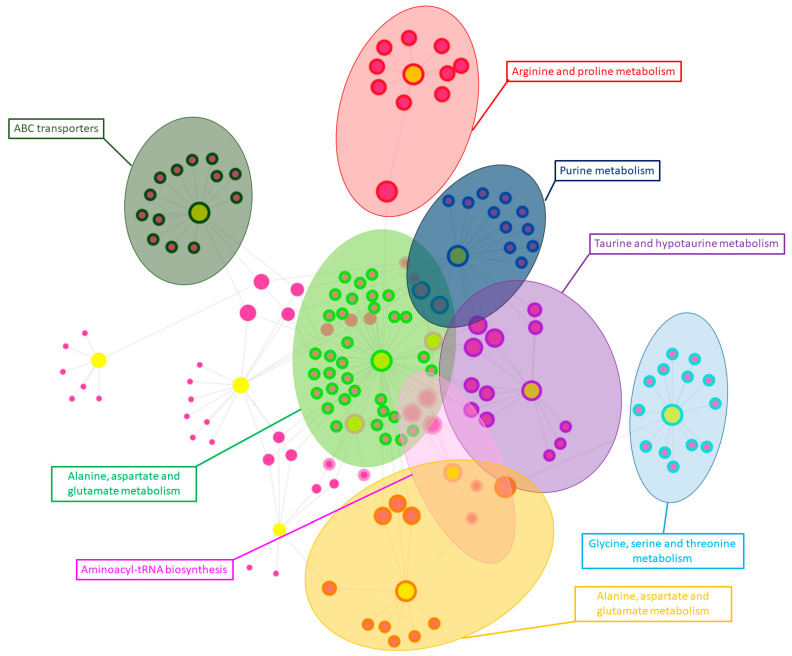
The multi-omics network created with OmicsNet. Yellow nodes represent metabolites, while salmon colored nodes represent proteins. Significant modules of connected metabolites and proteins are highlighted in different colors and annotated with their most significant top integrative KEGG pathways.

A knowledge-driven multi-omics network was created with OmicsNet using significantly altered proteins and metabolites between the control and *F. magna*-infected groups to provide a comprehensive visual representation. The multi omics-network was further searched by the InfoMap algorithm to identify densely connected modules, resulting in eight significant modules. These modules were subsequently searched and annotated with the top integrative KEGG pathways (Figure 10). In this way, the multi-omics network demonstrates the interplay among significant proteins and metabolites, highlighting altered pathways between the control and *F. magna*-infected groups across different omics layers.

## 4. Discussion

Red deer are economically and culturally significant wildlife and game species in Europe and worldwide. They are scientifically important for phylogenetic studies, particularly in relation to population decline and genetic diversity influenced by new settlers. In medicine, deer antlers are notable for their potential in organ regeneration and cancer studies. In addition, deer are studied for infectious and parasitic diseases that can affect humans and domestic animals. Liver fluke infections are considered a globally neglected disease with a significant impact on public health [37]. Fascioloidosis has significant implications for both wild and domestic animals, as *F. magna* parasitizes in the liver of infected hosts, causing macroscopic and microscopic lesions that can lead to severe liver damage and dysfunction.

### 4.1. Proteomics

The TMT-based proteomics approach was applied to determine differences in protein abundances between the *F. magna*-infected and control groups, resulting in 18 proteins with significantly different abundances. According to gene ontology, these proteins were linked to response to stimulus (e.g., immune response and response to stress), immune system processes, biological regulation, and cellular processes (e.g., cell activation, cell adhesion, and cell metabolic processes).

The central role of the host response to the parasite is evidenced by the significantly abundant proteins associated with the immune system, such as acute phase proteins and components of complement cascade.

Among the differentially abundant serum proteins, acute phase proteins (APPs) such as fibrinogen, alpha-1-acid glycoprotein, albumin, inter-alpha-trypsin inhibitor, and histidine-rich glycoprotein stand out. Despite the uniform nature of the acute phase response—a non-specific and complex reaction of an organism triggered by various stimuli including infection—there are numerous differences in acute phase characteristics between different animal species [38]. Studies on APP in cervids are relatively scarce. Fibrinogen has been shown to act as an APP; increased fibrinogen concentrations have been observed in sick reindeer [39], after an experimental infection of red deer with herpesvirus causing malignant catarrhal fever [40], in red deer following tuberculine testing [41], and after inoculation with *Yersinia pseudotuberculosis* [42]. In our study, fibrinogen chains (alpha, beta, and gamma) were among the top three identified proteins, with the highest increase in the *F. magna*-infected group, which could indicate an acute phase response due to chronic inflammation. An increased abundance of alpha-1-acid glycoprotein (AGP), previously unreported in red deer, was also found in the *F. magna*-infected group. Besides its immunomodulating effects, AGP binds and carries basic and neutral lipophilic drugs from both endogenous and exogenous sources [43].

Lower abundances of inter-alpha-trypsin inhibitor heavy chains H1 and H2 (ITIH1, ITIH2) were found in the *F. magna*-infected group compared to controls. Inter-alpha-trypsin inhibitors are a family of plasma serine protease inhibitors that play a role in the acute phase response and contribute to extracellular matrix stability by covalent linkage to hyaluronan. A previous study exploring the liver proteome in *F. magna*-infected red deer reported an alteration in different fibrosis-related proteins, suggesting the remodeling of the actin cytoskeleton due to the parasite [18]. Liver fibrosis occurs as a result of parasite migration through the liver and the continuous remodeling process, which is marked by the excessive deposition of extracellular matrix in the hepatic parenchyma. Lower abundances of ITIH1 and ITIH2 may contribute to reduced protease inhibitor activity, potentially preventing excessive protease-mediated liver injury. The rapid consumption of protease inhibitors limits the potentially deleterious effects of protease activation on endothelial and epithelial tissues, as reported in sepsis [44]. Additionally, lower abundances of fetuin-B, another plasma proteinase inhibitor, were also found in the *F. magna*-infected group.

The identification of complement-related proteins, such as complement component C8 and complement factor H, in the serum of red deer infected with *F. magna* supports the role of the complement cascade in the innate immune response to the pathogen. The complement system may activate platelets or induce biochemical and morphological changes in the endothelium, thereby enhancing coagulation and contributing to homeostasis in response to stimuli [45]. Our work further emphasizes this by highlighting platelet-associated pathways, namely platelet degranulation, response to elevated platelet cytosolic Ca^2+^, and platelet activation-, signaling-, and aggregation-enriched pathways. The coagulation system plays a crucial role in host–pathogen interactions and the host’s immune responses.

Another highlighted pathway in this study was the regulation of insulin-like growth factor (IGF) transport and uptake by IGF. Its expression in the vasculature is affected by interactions with cytokines, lipoproteins, growth factors, reactive oxygen species, and hemodynamic forces [46]. The roles of this pathway and the proteins involved, such as fibrinogen, albumin, apolipoprotein A1, and inter-alpha-trypsin inhibitor, suggest the importance of vascular interactions in the pathogenesis of fascioloidosis.

We believe that, on their own, none of the obtained proteins are likely specific enough as markers for *F. magna* infection in deer. However, this study suggests that fibrinogen and alpha-1-acid glycoprotein may be valuable as acute-phase proteins in deer and could be considered as indicators of inflammatory responses, while the roles of other proteins should be further investigated.

### 4.2. Metabolomics

An untargeted MS-based metabolomics analysis of *F. magna* infection in red deer resulted in the generation of a serum metabolic signature reflective of the pathogen.

Previous nuclear magnetic resonance (NMR)-based metabolomics studies of helminth infections revealed the following three major metabolic traits: alteration in amino acid metabolism, an infection-driven shift in lipid metabolism, and changes in metabolites associated with the gut microbiota [22]. Recently, four parasite–rodent models were used to investigate the metabolic profile of the host using NMR spectroscopy [47]. Additionally, the integrated response of tissue and biofluids in a rat host to the liver fluke *F. hepatica* was investigated in a comprehensive study utilizing NMR and multiplex cytokine markers [48]. Although altered metabolic signatures due to pathogen infections have been observed in rodent models, little is known about the metabolic fingerprints of infections in other mammals. More recently, metabolomic analysis and systematic biochemical tests on sheep infected with *F. hepatica* showed alterations in energy metabolism and nutrient deprivation [49].

An alteration in amino acid metabolism was one of the prominent findings in the *F. magna*-infected group, consistent with previous studies on helminth-infected animals [22,48,50]. Ten amino acids had higher intensities in the *F. magna* group, including essential amino acids such as valine, phenylalanine, methionine, leucine, and isoleucine. These amino acids are considered general markers for protein degradation, as variations in their levels are due to the catabolism of existing proteins resulting from tissue destruction, apoptosis, or autophagy [51]. In our case, we can hypothesize that the degradation of proteins is due to the presence of flukes, which cause damage to liver tissue and modulate energy requirements.

Two N-linked glycoproteins (N-acetylneuraminic acid and N-acetylglutamic acid) were found to be elevated in the *F. magna*-infected group, consistent with increased levels of N- and O-acetylated glycoproteins observed in the plasma of *Trypanosoma brucei brucei*- and *F. hepatica*-infected rodents [47]. These metabolites serve as ligands for cell adhesion molecules and play an important role in cell stability and direct interactions with pathogens and toxins [52,53]. Therefore, the increased intensities likely reflect an inflammatory response to *F. magna* infection in red deer.

Another significant metabolic pattern observed in the group infected with *F. magna* was related to energy metabolism. The host and parasite compete for available metabolic building blocks and energy resources, so the utilization of alternative metabolic pathways maintains the balance of host metabolism. Hence, the host’s adaptations to *F. magna* infection in energy metabolism are reflected in the serum metabolome. Higher intensities of methylmalonic acid, malate, pantothenic acid, malonate, citraconic acid, and citramalic acid were found in the serum of infected red deer. Malate is a metabolic intermediate in the citric acid cycle and is also important for the malate–aspartate shuttle system, which enables the transport of electrons across the membrane between the cytosol and the mitochondrial matrix [54]. Malonate acts as a competitive inhibitor of succinate dehydrogenase, the only enzyme involved in both the citric acid cycle and the electron transport chain. Methylmalonic acid, a derivative of malonic acid, serves as part of the anaplerotic reaction for the citric acid cycle. Pantothenic acid is a B5 vitamin required for the synthesis of coenzyme A, which is essential for cellular energy production and for the synthesis and breakdown of proteins, carbohydrates and lipids [54]. These changes in energy metabolism are also supported by an earlier proteomics study that found altered abundances of various enzymes involved in carbon metabolism, the citric acid cycle, and oxidative phosphorylation in the liver of red deer infected with *F. magna* [18]. The main biochemical effects of *S. japonicum* infection in hamsters included reduced levels of urinary citric acid cycle intermediates, including citrate and succinate, alongside increased levels of pyruvate, indicating stimulated glycolysis [55]. Similarly, in buffaloes infected with *F. gigantica*, the downregulation of metabolism-related processes in the liver was prominent across all time points [20].

Changes in metabolites related to energy metabolism, particularly in the citric acid cycle, support the hypothesis of a disruption of energy metabolism in fascioloidosis as a result of infection and the switch to alternative energy pathways. The parasite consumes large amounts of host glucose and has a greatly reduced lipid metabolism due to its inability to process beta-oxidation [56,57]. The modulation of energy metabolism in the host is further supported by increased protein degradation, as evidenced by an altered amino acid pattern and the increased intensity of methylhistidine, a marker for protein breakdown in skeletal muscle [54]. However, further and more detailed studies are needed to decipher the exact alternative pathways and the adaptive response of the host.

Changes in lipid metabolism are also evident in this study, with higher intensities of choline and its derivative betaine observed in the *F. magna*-infected group. These metabolites can be further converted into phosphocholine, a fundamental component of membrane synthesis, or utilized for producing polyunsaturated fatty acids like arachidonic acid, leading to the synthesis of pro-inflammatory eicosanoid mediators [58]. Choline is associated with the inflammatory host response to the parasite demonstrating anti-inflammatory activity by restraining excessive inflammation through the activation of the cholinergic anti-inflammatory pathway [59,60].

Higher glycerol-3-phosphate intensity in the *F. magna*-infected group further indicates increased lipid degradation and enhanced gluconeogenesis.

Several pathways related to transcription/translation, tRNA aminoacylation, and metabolites related to nucleotide derivatives were highlighted in this study. Inosine is a product of the intracellular conversion of adenosine by nucleotide-degrading enzymes secreted by the fluke [61,62]. The shift to inosine in the *F. magna*-infected group suggests an attenuated inflammatory response that suppresses pro-inflammatory cytokines such as IFN-γ, TNF-α, and IL-12, ultimately prolonging fluke survival [63].

### 4.3. Integration of Proteomics and Metabolomics Data

The integrated omics approach based on the parallel and integrated assessment of metabolite and protein profiles enabled the extraction of biochemical pathways directly and indirectly associated with *F. magna* infection in red deer.

The most highlighted functional annotations from the integrated proteomics and metabolomics data were related to amino acid metabolism. Despite the fact that amino acids always appear on the list of metabolites affected by infection, several similarities were observed in helminth-infected animals. Higher levels of essential amino acids, such as valine, are consistent with the findings from *Schistosoma* and *Fasciola* models [48,64], suggesting that the parasite disrupts the insulin-stimulated uptake of essential amino acids by tissues, in order to benefit from an increased pool of this important carbon and energy source in the blood. Furthermore, taurine is another amino acid metabolite frequently affected by helminth infections [48,64]. Taurine and hypotaurine metabolism are closely linked to glutathione metabolism, which plays a role in the protection against oxidative stress [65]. Previous studies have reported reduced activity of antioxidant enzymes due to parasite infections. Infection with *F. hepatica* reduces antioxidant capacity in humans and rats [66,67,68]. Alterations in the components and pathways associated with oxidative stress were also found in red deer and wild boar infected with *F. magna* [17,18], indicating that infection with *F. magna* is accompanied by changes in antioxidant capacity.

One of the top functional annotations of the integrated proteomics and metabolomics data was related to ATP-binding cassette (ABC) transporters. ABC transporters form a ubiquitous superfamily of integral membrane proteins responsible for the ATP-driven translocation of various substrates across membranes, ranging from ions to macromolecules [69]. ABC transporters are associated with various host–pathogen interactions and have been linked to reduced drug susceptibility in parasitic helminths [70].

Several flukicide drugs are available, but the treatment of choice for both juvenile and adult stages in final hosts is the benzimidazole, triclabendazole (TCBZ). The control of liver fluke infections has predominantly relied on treatment with anthelmintic drugs in endemic areas. As part of the animal disease control program, medicated baits containing triclabendazole have been made available in the free-ranging deer population [71]. Unfortunately, the intensive use of strong flukicides has led to the development of resistance. Reports of TCBZ-resistant *F. hepatica* populations in cattle, sheep, and humans, could undermine fluke control and necessitate research into new treatment options [72,73,74]. Several in vitro and in vivo experiments have elucidated the role of the CYP450 system in resistance and susceptibility to antiparasitic drugs [75,76,77]. As a cellular stress response to parasite presence, the host’s response includes the upregulation of a suite of protective and detoxifying genes, including ABC transporters and cytochrome P450 detoxification proteins [78]. The enzymes involved in the metabolism of xenobiotics showed significant changes in red deer and wild boar infected with *F. magna*, as well as in sheep infected with the fluke *F. hepatica* [17,18,79]. Additionally, different drugs’ action pathways were significantly affected in our metabolomics pathways’ enrichment analysis. Therefore, our results support the hypothesis of the involvement of these pathways and proteins in the development of drug resistance, suggesting a need for further research.

Purine metabolism and aminoacyl-tRNA biosynthesis were also emphasized in this study. Purine metabolism synthesizes key structural elements for nucleic acid synthesis, as well as energy metabolism molecules, such as coenzymes, allosteric modulators, energy intermediates, and intracellular and extracellular messengers. Extracellular nucleosides and nucleotides are involved in various cellular responses, including proliferation, migration, differentiation, and the secretion of growth factors and inflammatory mediators [80]. Aminoacyl-tRNA synthetases (ARSs) are a vital and ubiquitously present family of enzymes essential for protein synthesis. However, the growing body of evidence shows that ARS are involved in a variety of physiological and pathological processes beyond translation [81]. ARSs participate in the maturation, transcription, activation, and recruitment of immune cells, functioning as regulators and signaling molecules in various infectious diseases. Considering the above, exploring the biological functions of these pathways in an immune context represents a promising field for future studies.

### 4.4. Strengths and Limitations of the Study

Studying wild animals is often more complex due to challenges in study design, monitoring, data collection, and interventions in free-ranging populations and open habitats. As we studied naturally infected free-ranging deer with varying stages of infection and individual variability, caution is necessary for interpretation, thereby representing potential limitations of the study.

The strengths of this research include the use of two complementary, up-to-date omics approaches based on high-resolution mass spectrometry. A major challenge in untargeted metabolomics is metabolite identification, influenced by technological limitations, including the analytical coverage of the employed platform, biases favoring the detection of more abundant molecules, and differing fragmentation patterns of the same metabolite. While the small sample size may be considered a limitation, the strong effects of infection were sufficient to create a clear picture of the dominant proteins and metabolites associated with the disease.

## 5. Conclusions

Public health programs and emerging infectious disease surveillance must prioritize diseases at the wildlife–livestock interface, especially zoonotic ones. Understanding wildlife and their roles is crucial for grasping the epidemiology and ecology of these diseases. Understanding host–parasite interactions in wild animals is essential for predicting the impact of both the spread of existing parasites and the emergence of new ones on population management and dynamics.

In conclusion, we observed a pattern of significantly altered proteins and metabolites referring to the inflammatory host response and energy metabolism modulation in deer fascioloidosis. Our findings demonstrated that infection with the giant liver fluke *F. magna* in red deer was associated with changes in amino acid metabolism, energy metabolism, lipid metabolism, immune system, and related biochemical pathways. This study provides a global overview of the serum proteome and metabolome in response to *F. magna* infection in red deer, highlighting new targets for further investigation. The identification of proteins, metabolites and associated biological pathways represents a valuable contribution to the understanding of host–parasite interactions and may enhance current tools for more effective liver fluke control.

## Figures and Tables

**Table 1:** Proteins with significantly differential abundances between control and *F. magna*-infected red deer (*Cervus elaphus*) identified and quantified using a tandem mass tag (TMT) proteomics approach in the serum.

Accession	Gene Name	Description	*p* Value	FDR	log2FC
A0A212DAA2	FGA	Fibrinogen alpha chain	1 × 10^−5^	0.0002	1.380
A0A212D7J2	FGB	Fibrinogen beta chain	1 × 10^−5^	0.0002	1.296
A0A212D8V0	FGG	Fibrinogen gamma chain	2 × 10^−5^	0.0003	0.940
A0A212D4K0		Histone H4	1 × 10^−2^	0.0440	0.860
A0A212CNC6	ORM1	Alpha-1-acid glycoprotein	1 × 10^−5^	0.0002	0.727
A0A212D5P0	ALB	Albumin	8 × 10^−3^	0.0373	−0.173
A0A212CG89	C8A	Complement C8 alpha chain	6 × 10^−3^	0.0301	−0.188
A0A212C922	ITIH1	Inter-alpha-trypsin inhibitor heavy chain H1	1 × 10^−2^	0.0519	−0.190
A0A212CDE1	LOC506828	Uncharacterized protein (BLAST: Pregnancy zone protein)	3 × 10^−3^	0.0183	−0.221
A0A212CQC9	CFH	Complement factor H	4 × 10^−3^	0.0225	−0.228
A0A212CC12	ITIH2	Inter-alpha-trypsin inhibitor heavy chain 2	1 × 10^−3^	0.0105	−0.241
A0A212CIM7	HRG	Histidine-rich glycoprotein	1 × 10^−2^	0.0519	−0.309
A0A212CMY9	IGHM	Ig-like domain-containing protein	1 × 10^−2^	0.0430	−0.321
A0A212CIC4	FETUB	Fetuin-B	3 × 10^−3^	0.0183	−0.321
A0A212DHP9	APOA1	Apolipoprotein A-I	2 × 10^−3^	0.0183	−0.374
A0A212CI17	CD5L	CD5 molecule like	8 × 10^−3^	0.0374	−0.432
A0A212CQ10	CFH	Complement factor H	3 × 10^−3^	0.0183	−0.434
A0A212D5R7	JCHAIN	Joining chain of multimeric IgA and IgM	7 × 10^−4^	0.0092	−0.447

**Table 2:** A list of the identified and significantly changed metabolites in the serum of red deer infected with *F. magna* versus control red deer, obtained using the untargeted LC-MS metabolomics approach.

Peak ID	Name	Mass	RT	*p* Value	FDR	log2(FC)
1447	Methylmalonic acid	117.0196	700.59	4 × 10^−8^	1.25 × 10^−6^	4.87
72	Isonicotinic acid	124.0394	436.77	2 × 10^−3^	0.004722	4.03
42	4-Methoxybenzyl propanoate	258.1101	646.48	6 × 10^−7^	8.05 × 10^−6^	3.71
1551	L-Aspartate	132.0305	690.35	2 × 10^−5^	9.79 × 10^−5^	3.67
1497	Inosine	267.074	554.37	2 × 10^−3^	0.004966	3.43
1441	Malate	133.0145	734.56	2 × 10^−10^	1.92 × 10^−8^	3.38
1855	Glycerol 3-phosphate	171.0068	646.54	6 × 10^−7^	8.49 × 10^−6^	3.35
298	Imidazole-4-acetate	127.0502	584.57	2 × 10^−3^	0.004722	3.25
6	Inosine	269.0881	555.54	1 × 10^−3^	0.004086	3.18
95	2-Hydroxyadenine	152.0568	634.21	8 × 10^−8^	2.15 × 10^−6^	3.08
364	Pantothenic acid	220.1179	463.76	2 × 10^−7^	4.23 × 10^−6^	2.76
1844	Pantothenic acid	218.1038	463.33	2 × 10^−7^	4.55 × 10^−6^	2.74
20	Choline	104.107	1037.39	3 × 10^−7^	5.93 × 10^−6^	2.47
1433	Pseudouridine	243.0627	522	3 × 10^−8^	8.99 × 10^−7^	2.39
1948	Citraconic acid	129.0197	700.79	5 × 10^−8^	1.52 × 10^−6^	2.32
199	Taurine	126.022	729.49	4 × 10^−7^	7.08 × 10^−6^	2.26
2256	Cytidine	242.0789	597.61	6 × 10^−3^	0.012562	2.15
1671	Ureidopropionic acid	131.0465	709.18	1 × 10^−3^	0.003059	2.15
1486	Taurine	124.0076	730	2 × 10^−7^	4.20 × 10^−6^	2.06
363	3-Deoxy-D-glycero-D-galacto-2-nonulosonic acid	310.1132	647.3	5 × 10^−5^	0.000255	1.94
1561	N-Acetylneuraminic acid	308.0994	628.17	2 × 10^−5^	0.000128	1.93
1683	N-Acetylglutamic acid	188.0569	654.1	1 × 10^−4^	0.000591	1.60
50	L-Glutamate	148.0604	674.5	2 × 10^−6^	1.74 × 10^−5^	1.54
83	3-Dehydroxycarnitine	146.1176	616.16	3 × 10^−5^	0.000162	1.49
1573	Galactonic acid	195.0514	655.33	1 × 10^−5^	9.32 × 10^−5^	1.38
1458	L-Glutamate	146.0462	674.96	2 × 10^−6^	1.75 × 10^−5^	1.38
1576	L-Alanine	88.0406	688.94	5 × 10^−5^	0.000248	1.04
682	Citramalic acid	190.0709	653.62	1 × 10^−3^	0.002955	0.90
2455	Malonate	103.0039	727.57	5 × 10^−4^	0.001558	0.82
5	Betaine	118.0862	557.5	4 × 10^−5^	0.000196	0.78
43	3-Methylhistidine	170.0924	610.68	3 × 10^−5^	0.000158	0.73
1687	Pterolactam	114.0563	618.23	8 × 10^−5^	0.000381	0.71
24	L-Proline	116.0706	618.02	1 × 10^−5^	9.31 × 10^−5^	0.69
1814	3-Methylhistidine	168.0782	611.63	1 × 10^−6^	1.12 × 10^−5^	0.67
282	L-Serine	106.0499	730.75	1 × 10^−2^	0.02085	0.54
1657	L-Phenylalanine	164.0721	515.76	2 × 10^−4^	0.000915	0.50
195	L-Methionine	150.0584	573.73	4 × 10^−3^	0.008503	0.49
57	L-Citrulline	176.1029	719.55	2 × 10^−2^	0.032129	0.48
1599	L-Leucine	130.0876	550.18	7 × 10^−4^	0.002242	0.46
1964	L-Valine	116.072	555.79	2 × 10^−4^	0.000882	0.39
1565	L-Leucine	130.0876	540.2	7 × 10^−3^	0.014649	0.36
55	L-Isoleucine	132.1019	540.6	5 × 10^−3^	0.010758	0.33
70	D-Alloisoleucine	132.1019	560.86	1 × 10^−2^	0.021533	0.32
2459	L-Kynurenine	207.0779	548.26	9 × 10^−3^	0.018014	-0.48

RT—retention time. FC—fold change.

## Data Availability

The original contributions presented in the study are included in the article/Supplementary Materials, further inquiries can be directed to the corresponding author.

## Supplementary Materials

The following supporting information can be downloaded at: https://www.mdpi.com/article/10.3390/pathogens13110922/s1, Supplementary data: Supplemental Table S1. List of all identified and quantified proteins in serum samples. Supplemental Table S2. Reactome pathways enriched from serum proteins significantly differential in abundance between control and F. magna-infected groups. Supplemental Table S3. Peak intensity table with mass, retention time and annotations of features identified by untargeted metabolomics approach in the serum of control and F. magna-infected red deer. Supplemental Table S4. List of all identified features on the basis of the mass/retention time matched to known standards in the PiMP software. Supplemental Table S5. Pathway enrichment of the significant metabolites identified by untargeted metabolomic approach between control and *F. magna*-infected group based on 3694 metabolites and lipid pathways from RaMP-DB.
